# Treatment of Lupus

**Published:** 1879-03-20

**Authors:** 


### *TREATMENT OF LUPUS.*

John C., aged about forty, some two years ago had what he thought a wart upon the pinna of the ear. This wart proving troublesome he was in the habit of picking at it, until it became an ulcer; he then applied many home-made salves, but failing to heal the sore, applied to me for treatment. I was satisfied that I had a case of lupus to contend with, and told him it would be necessary to burn it; this he would not agree to until I had given him several ointments, such as carbolic, ung. hdyg., ammonia, etc., to try and heal it. These having failed, as I anticipated, he consented to submit to any treatment I might deem advisable. The ulcer now extended over the greater portion of the upper part of the ear and the adjacent part of the head. On the 20th of October, 1878, I applied a paste made of chloride zinc one part, and flour three parts, as advised by Erichsen, and sent him home, some five miles, with some morphia powders to take during the night, to relieve pain. On the morning of the 21st I visited him. He had not taken the morphia, saying the pain was not so severe as to require it. The whole of the ear and surrounding parts were much swollen and inflamed. Ordered flaxseed poultice; the swelling and inflammation gradually subsided, and on the 26th the whole of the slough or eschar caused by the paste had come away, leaving a healthy granulating surface. I now ordered the application of the following ointment: Ungt. hydrarg. nit., ʒj, cosmoline, ℥j, recommended by Dr. G. McConnell, *Med. and Surg. Reporter*, Sept. 21st, 1878. The sore healed rapidly, and in ten days from application of zinc paste the man was at work.

The main points of interest in my case are, 1st, the slight amount of pain, the patient walking five miles after application of the paste, and not taking the morphia with which he had been supplied, at any time during treatment, though the paste was kept on some twelve hours, and set up considerable inflammation. 2d. The short time required to effect a radical cure of a lupus of two years' standing. The object in treatment is to stimulate the vessels supplying the parts, so as to substitute active healthy action for the sluggish, indolent condition always found in lupus; therefore your paste should be sufficiently strong and applied long enough to set up considerable inflammation, and to penetrate through the diseased to the healthy tissues, so that when your eschar comes off it may take the whole of the diseased part with it, otherwise you may have to make more than one application.—*Dr. Alexander, in Med. and Surg. Reporter.*

